# LPS-Binding Protein and IL-6 Mark Paradoxical Tuberculosis Immune Reconstitution Inflammatory Syndrome in HIV Patients

**DOI:** 10.1371/journal.pone.0081856

**Published:** 2013-11-28

**Authors:** Odin Goovaerts, Wim Jennes, Marguerite Massinga-Loembé, Ann Ceulemans, William Worodria, Harriet Mayanja-Kizza, Robert Colebunders, Luc Kestens, the TB-IRIS Study Group

**Affiliations:** 1 Department of Biomedical Sciences, Institute of Tropical Medicine, Antwerp, Belgium; 2 Department of Biomedical Sciences, University of Antwerp, Antwerp, Belgium; 3 Centre de Recherches Médicales de Lambaréné (CERMEL), Albert Schweitzer Hospital, Lambarene, Gabon; 4 Institut für Tropenmedizin, Universität Tübingen, Tübingen, Germany; 5 Department of Medicine, Mulago Hospital, Kampala, Uganda; 6 Infectious Diseases Institute, Makerere University College of Health Sciences, Kampala, Uganda; 7 Infectious Diseases Network for Treatment and Research in Africa (INTERACT), Kampala, Uganda; 8 Department of Clinical Sciences, Institute of Tropical Medicine, Antwerp, Belgium; 9 Epidemiology and Social Medicine, University of Antwerp, Antwerp, Belgium; Kings College London, United Kingdom

## Abstract

**Background:**

Tuberculosis-associated immune reconstitution inflammatory syndrome (TB-IRIS) remains a poorly understood complication in HIV-TB co-infected patients initiating antiretroviral therapy (ART). The role of the innate immune system in TB-IRIS is becoming increasingly apparent, however the potential involvement in TB-IRIS of a leaky gut and proteins that interfere with TLR stimulation by binding PAMPs has not been investigated before. Here we aimed to investigate the innate nature of the cytokine response in TB-IRIS and to identify novel potential biomarkers.

**Methods:**

From a large prospective cohort of HIV-TB co-infected patients receiving TB treatment, we compared 40 patients who developed TB-IRIS during the first month of ART with 40 patients matched for age, sex and baseline CD4 count who did not. We analyzed plasma levels of lipopolysaccharide (LPS)-binding protein (LBP), LPS, sCD14, endotoxin-core antibody, intestinal fatty acid-binding protein (I-FABP) and 18 pro-and anti-inflammatory cytokines before and during ART.

**Results:**

We observed lower baseline levels of IL-6 (p = 0.041), GCSF (p = 0.036) and LBP (p = 0.016) in TB-IRIS patients. At IRIS event, we detected higher levels of LBP, IL-1RA, IL-4, IL-6, IL-7, IL-8, G-CSF (p ≤ 0.032) and lower I-FABP levels (p = 0.013) compared to HIV-TB co-infected controls. Only IL-6 showed an independent effect in multivariate models containing significant cytokines from pre-ART (p = 0.039) and during TB-IRIS (p = 0.034).

**Conclusion:**

We report pre-ART IL-6 and LBP levels as well as IL-6, LBP and I-FABP levels during IRIS-event as potential biomarkers in TB-IRIS. Our results show no evidence of the possible contribution of a leaky gut to TB-IRIS and indicate that IL-6 holds a distinct role in the disturbed innate cytokine profile before and during TB-IRIS. Future clinical studies should investigate the importance and clinical relevance of these markers for the diagnosis and treatment of TB-IRIS.

## Introduction

Over one third of the 33 million people living with HIV are co-infected with tuberculosis (TB) [1]. While the rollout of antiretroviral therapy (ART) has increasingly contributed to halt HIV disease progression and to reduce the risk of opportunistic infections, complications still occur. During successful ART, up to 25% of HIV-TB co-infected patients paradoxically develop worsening symptoms of TB, despite effective initial response to concurrent TB treatment [2]. This complication has been named paradoxical tuberculosis-associated immune reconstitution inflammatory syndrome (TB-IRIS) [3] and may require hospitalization or additional immune suppressive therapy [4]. TB-IRIS typically develops within the first 2 months after starting ART (early-onset TB-IRIS), with the majority of cases occurring before 1 month [5]. As TB-IRIS is often difficult to distinguish from other complications, there is an urgent need for reliable laboratory markers to predict and identify this syndrome [6].

Although several risk factors such as low CD4 count, high TB-antigen burden and short interval between initiation of TB treatment and ART are well established [7,8], the actual pathogenesis of TB-IRIS remains to be elucidated. TB-IRIS is characterized by tissue-destructive inflammation when CD4+ T-cells are being replenished [9] and might thus involve an amplified immune reaction to TB bacilli or their residual antigens [10]. Many studies have therefore focused on the potential role of T-cells and a polarized T-helper 1 cell response in TB-IRIS [11–13]. In contrast, recent opinions have arisen that implicate cells of the innate immune system in TB-IRIS pathogenesis [14] or indeed disturbances in the interplay between the innate and adaptive immune system [9] which could lead to inflammatory conditions upon ART initiation. 

Inflammation in TB-IRIS coincides with elevations in a plethora of cytokines, referred to as the cytokine storm since it was first observed [11]. Most studies of IRIS to date have focused on pro- and anti-inflammatory cytokines during the IRIS event [12,13,15–17]. Interestingly, several studies noted a role for cytokines of myeloid or dual myeloid/lymphoid origin, like IL-6 and TNFα, in the ongoing inflammation [17]. Other plasma markers were less consistently reported across studies, possibly as a result of variations in size and origin of the study populations, comparisons that are unmatched for baseline CD4 count, or inclusion of IRIS cases related to other, non-TB pathogens. It is therefore still unclear which cells and cytokines lie at the basis of TB-IRIS and its inflammation.

The immune reaction and inflammation in response to TB typically involves stimulation of toll-like receptors (TLR) by antigens such as lipoarabinomannan (LAM) grouped under the name pathogen associated molecular patterns (PAMPs). Interestingly, a higher level of pre-ART urinary LAM has been reported in TB-IRIS patients [7]. This finding, along with reports of elevated C-reactive protein (CRP) prior to ART [18,19] and during IRIS event [20,21], supports a possible role for the TLR-pathway in TB-IRIS. However, the potential involvement of gut derived bacterial PAMPs such as LPS and proteins that regulate TLR stimulation by binding PAMPs remains largely unexplored. LPS enters the blood through a phenomenon known as a “leaky gut”, characterized by increased intestinal permeability, which is associated with immune activation in HIV disease [22]. LPS-binding protein (LBP) and soluble CD14 (sCD14) are able to regulate this antigenic stimulation by binding LPS and other PAMPs [23–27] and could therefore influence TB-IRIS inflammation.

In this study we aimed to find biomarkers for early-onset TB-IRIS in one of the largest TB-IRIS cohorts described to date. We hypothesized that a more pronounced presence of PAMPs from a leaky gut such as LPS could contribute to the cytokine storm in TB-IRIS in a non-TB related manner and that this effect is regulated by plasma proteins that bind PAMPs, which have not been investigated in TB-IRIS before. We report lower plasma levels of LBP before ART initiation but higher levels of LBP during IRIS event. Similarly, the innate cytokine profile showed lower levels before ART and higher levels during TB-IRIS, with IL-6 holding a dominant role. Our results support the theory that dysfunctions in the innate immune system make a large contribution to TB-IRIS pathogenesis. 

## Materials and Methods

### Study population

Patients from a prospective observational study at Mulago Hospital, Kampala, Uganda, were studied as described previously [7,28,29]. We recruited HIV-TB co-infected adults who were receiving treatment for active TB infection for no more than 2 months and were eligible for ART. After enrollment, all patients were started on a non-nucleoside reverse transcriptase inhibitor-based ART according to Ugandan national guidelines. Including adherence preparation, which took 2 to 3 visits to the clinic, the median interval from starting TB treatment to starting ART for all patients was 6 weeks. Patients were followed up for a minimum of 3 months to monitor paradoxical TB-IRIS development. Patients who did not develop IRIS-related symptoms for a minimum of 3 months served as controls. Plasma samples were collected before initiation of ART (baseline) and at 2 weeks, 4 weeks, 3 months, and 6 months after starting ART. Patients who developed TB-IRIS had an extra blood sample taken when diagnosed with inflammatory symptoms during ongoing IRIS event. 

### Patient selection and matching

The large majority of TB-IRIS patients from our cohort developed an IRIS event within the first month of ART. To limit heterogeneity amongst TB-IRIS patients, we excluded all patients who developed TB-IRIS later than 1 month. This homogeneous selection of patients reduces potential bias due to different cytokine kinetics and immunopathology between early- and late-onset TB-IRIS. TB-IRIS patients included in this study developed IRIS between 4 and 28 days on ART and were paired with controls at their closest corresponding time points. We thus matched IRIS events, sampled during ongoing inflammation, from 4 to 20 days on ART with control samples taken after 2 weeks on ART and IRIS events from 21 to 28 days on ART with control samples taken after 1 month on ART. TB-IRIS patients were also matched 1 by 1 with controls for sex, baseline CD4 count and age. After matching for sex, we secondly prioritized matching patients with +/- 15 CD4 cells/mm³. Thirdly, patients were matched for age with ≤ 10 years difference. 

### Definitions

*Mycobacterium tuberculosis* infection was diagnosed according to the TB/HIV WHO guidelines [30]. Investigations to confirm TB infection included: clinical examination, chest X-rays and abdominal ultrasounds, sputum smear microscopy for acid-fast bacilli and mycobacterial culture of sputum, aspirate or effusion if available. TB-IRIS cases were classified by a committee of two co-authors (RC and WW) after reviewing all suspected TB-IRIS cases evaluated by the study physicians according to the International Network for the Study of HIV-associated IRIS (INSHI) clinical case-definition [3]. TB-IRIS was diagnosed and patients were subsequently sampled when they presented with at least 1 major criterion, such as enlarged lymph nodes, or 2 minor criteria, such as fever and cough. Patients who developed TB-unrelated types of IRIS were excluded from the analysis. 

### Plasma analysis

Venous blood was drawn into EDTA tubes and plasma was separated from cells by centrifugation and stored at -80°C. Plasma concentrations of LBP, sCD14 and markers of a leaky gut, i.e. endotoxin-core antibody IgG (EndoCab) and intestinal fatty-acid-binding protein (I-FABP), were determined by ELISA (HyCult biotechnology BV, Uden, The Netherlands). Dilutions were 2000x (LBP), 100x (sCD14), 100x (EndoCab) and 2x (I-FABP) for each protein respectively. When enough plasma was available, lipopolysaccharide (LPS) levels were determined by using the kinetic limulus amebocyte lysate assay (Kinetic-QCL, Lonza) according to the manufacturer’s instructions. 

Plasma concentrations of 18 cytokines and chemokines were determined in duplicate using Bio-Plex™ human cytokine assay kits (Bio-Rad Laboratories NV-SA, Nazareth, Belgium) according to the manufacturer’s instructions. A range of cytokines from myeloid or lymphoid origin that could play a role in TB-IRIS were selected. These included IL-1RA, IL-2, IL-4, IL-6, IL-7, IL-8, IL-9, IL-10, IL-12p70, IL-17, interferon gamma (IFNγ),granulocyte-colony stimulating factor (G-CSF), granulocyte-monocyte colony stimulating factor (GM-CSF), Eotaxin-1 (CCL11), macrophage inflammatory protein 1β (CCL4), RANTES (CCL5), tumor necrosis factor alpha (TNFα) and interferon gamma-induced protein 10 (CXCL10). The Bio-Plex™ array reader and Manager 5.0 software were used to analyze cytokine concentrations using a weighted five-parameter logistic curve-fitting method. Results below limit of detection (LOD) were given the value LOD/2.

### Ethical considerations

The study was approved by the institutional review board of the Institute of Tropical Medicine of Antwerp, the Ethics Committees of the Faculties of Medicine of the University of Antwerp and the University of Makerere, the Research Committee of the Mulago Hospital and the Uganda National Council of Science and Technology. Written informed consent was obtained from all study participants.

### Statistical analysis

Statistics were performed using SPSS software (version 17.0), R software (version 2.15.0) or GraphPad Prism (version 5) with significance level set at p <0.05. Differences between paired patients and changes in concentration over time within each patient were analyzed using the Wilcoxon signed-rank test for paired data. Variables with >70% undetectable measurements were transformed into dichotomous variables and analyzed with a McNemar test for nominal data. No correction for multiple testing was applied as this is a hypothesis driven study with the aim to generate new biomarkers of TB-IRIS for further research [30,31]. Conditional multivariate logistic regression was used to determine the strongest cytokine predictors. Cytokines were included in multivariate models if P values from Wilcoxon signed-rank or McNemar tests were <0.05 and stepwise elimination were applied to determine significant independent effects. 

## Results

### Study population

From a prospective study population of 254 HIV-TB co-infected patients who were started on ART at Mulago Hospital in Kampala in Uganda, 53 patients developed paradoxical TB-IRIS. Thirteen TB-IRIS patients were excluded, including 6 who developed IRIS later than 1 month (range: 42-84 days) on ART, 5 who had inadequate samples and 2 who presented with other co-infections in addition to TB. The remaining 40 TB-IRIS patients were paired with 40 HIV-TB co-infected controls who did not develop TB-IRIS, matched for sex, baseline CD4 count and age (table 1). A median difference of 1 day (interquartile range (IQR): 0 - 4) was observed for time on ART prior to sampling of IRIS event or corresponding time point between patients and controls, which was statistically significant. TB-IRIS patients and controls did not significantly differ with regards to baseline levels of CRP or interval between TB- and HIV-treatment initiation. All patients with available HIV viral loads showed a significant decrease in HIV viral load upon ART and no significant differences at either baseline or IRIS event were observed between the two groups

**Table 1:** Characteristics of TB-IRIS patients and matched controls.

**Variables**	**TB-IRIS (n=40)**	**Controls (n=40)**	**p^a^**
**Characteristics Baseline**			
Sex male n (%)	23 (58)	23 (58)	1.000
Age (years)	35 (31-42)	38 (32-42)	0.320
CD4 (cell/mm^³^)	21 (10-54)	24 (15-54)	0.549
Days between TB treatment and ART**^*b*^**	31 (24-58)	46 (30-62)	0.291
CRP (mg/L)	10.9 (5.62-28.80)	21.14 (5.77-27.39)	0.614
Temperature (°C)	36.3 (36.0 - 36.7)	36.4 (35.8 - 36.6)	0.742
Viral Load (Log copies/ml)	5.6 (5.3-5.8)	5.5 (5.2-5.9)	0.719
**Characteristics TB-IRIS event**			
Days since start of ART	14 (12-14)	14 (14-18)	**<0.001**
Viral Load (Log copies/ml)**^*c*^**	3.4 (3.1-3.4)	3.4 (3.3-3.8)	0.158
Temperature (°C)	37.8 (37.1-38.2)	36.3 (35.8-36.5)	**<0.001**
Weight gain since start ART (kg)	0.0 (-2.5-+1.0)	0.5 (-1.0-+2.0)	0.194

Values are shown as median values with interquartile range. Due to missing data and pairwise exclusion the number of patients may vary between 30 and 40 unless stated otherwise. Level of significance was set to p < 0.05. **^*a*^**Wilcoxon signed-rank test. **^*b*^**Number of days between the start of TB therapy and initiation of ART. ***^c^***n = 14.

### Markers of a leaky gut prior to and during ART

**Figure 1:**
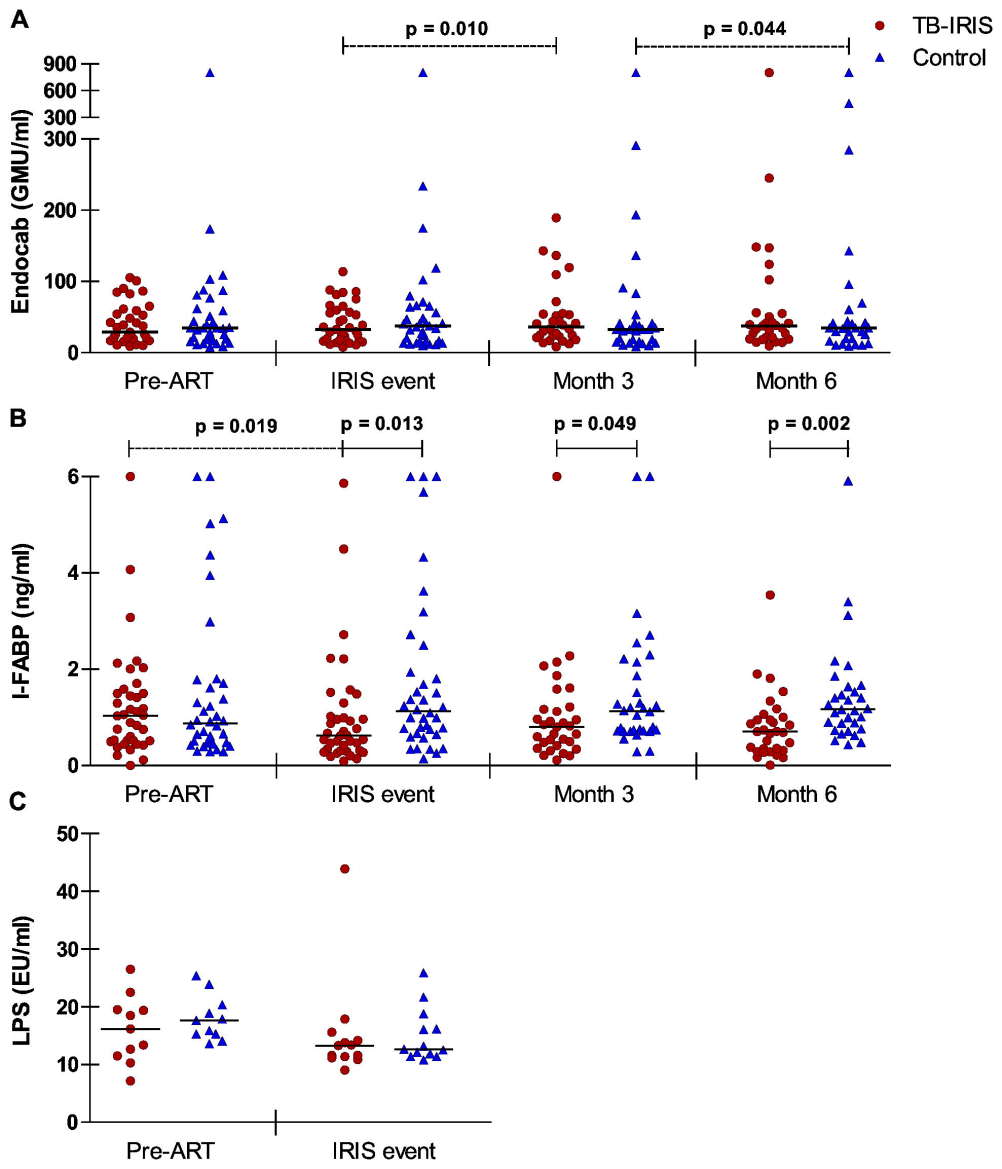
Markers of a leaky gut in TB-IRIS patients and controls during follow-up. Horizontal lines represent median plasma levels for each patient group at each time point. A Wilcoxon signed-rank test was used to calculate p values. The level of significance was set to p < 0.05. Dotted lines indicate significant changes over time per patient-group and full lines indicate significant differences between matched patients. (A) EndoCab. Number of patients in each group during consecutive time points is 35, 35, 30 and 29 respectively. (B) I-FABP. Number of patients in each group during consecutive time points is 38, 38, 32 and 31 respectively. (C) LPS. Number of patients in each group during consecutive time points is 11 and 13 respectively. GMU, IgG median units; EU, endotoxin units.

Increased intestinal permeability could lead to a more pronounced presence of PAMPs in the bloodstream. We assessed presence of LPS and damage to the intestinal epithelium by analyzing plasma levels of EndoCab and I-FABP in TB-IRIS patients and controls at baseline, IRIS event or corresponding time point and at 3 and 6 months on ART (Figure 1A & B). Additionally, when enough plasma was available, we analyzed LPS levels in TB-IRIS patients and controls at baseline and IRIS event or corresponding time point (Figure 1C). We observed no differences pre-ART between study groups. We did however observe a significant decline in I-FABP levels in TB-IRIS patients between pre-ART and IRIS event (p = 0.019) but not in controls. At IRIS event or corresponding time point, I-FABP levels were significantly lower in TB-IRIS patients compared to controls (p = 0.013), and they remained so at month 3 (p = 0.049) and month 6 (p = 0.002) on treatment. No differences between groups were observed for LPS nor for its antibody EndoCab.

### PAMP binding plasma proteins prior to and during ART

**Figure 2:**
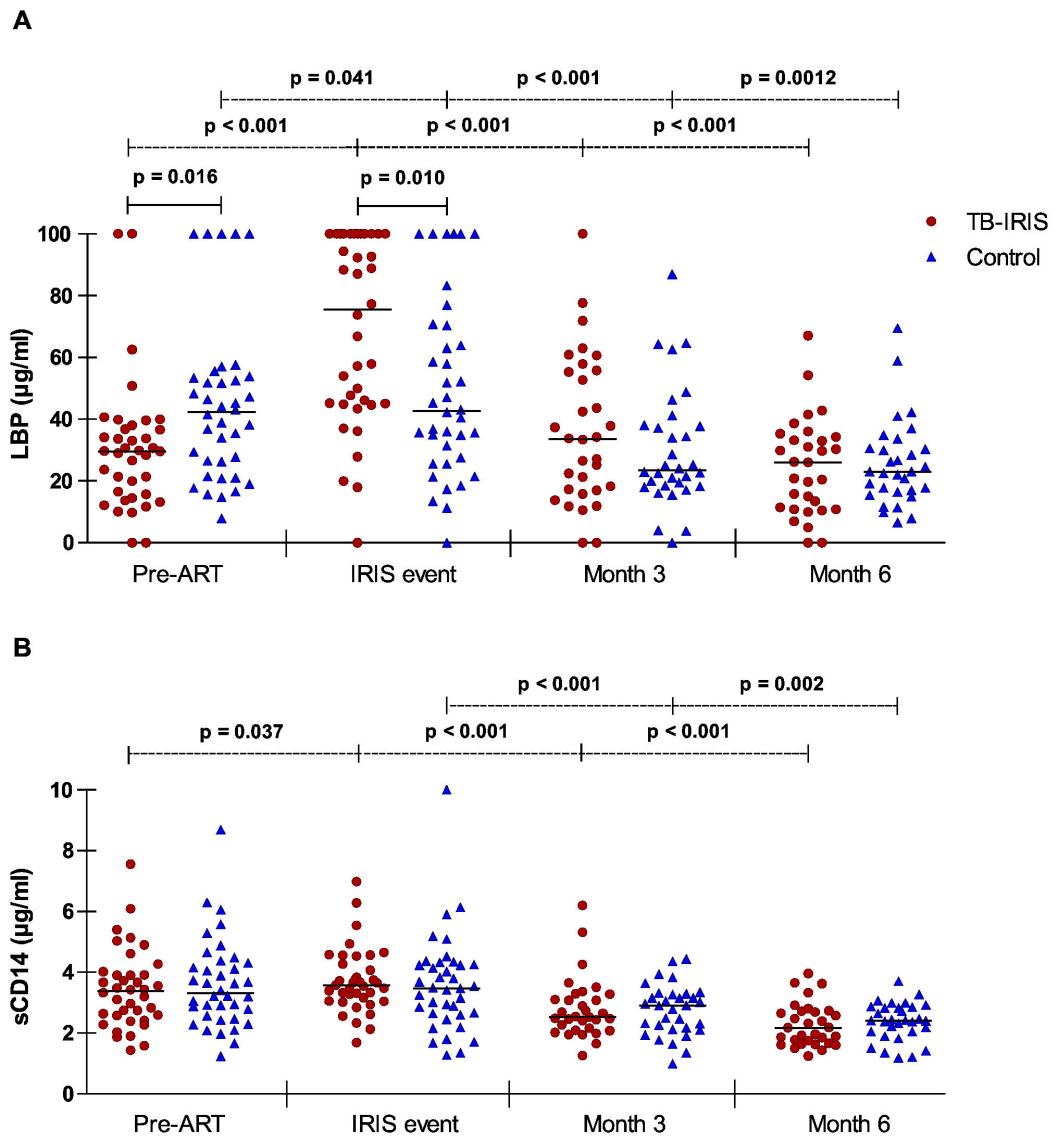
LBP and sCD14 plasma levels in TB-IRIS patients and controls during follow-up. Horizontal lines represent median levels of LBP (A) and sCD14 (B) for each patient group at each time point. A Wilcoxon signed-rank test was used to calculate p values. The level of significance was set to p < 0.05. Dotted lines indicate significant changes over time per patient-group and full lines indicate significant differences between matched patients. Number of patients in each group during consecutive time points is 38, 38, 32 and 31 respectively.

Proteins that bind PAMPs have the ability to interfere with inflammatory processes. To evaluate the possible importance of these proteins, we evaluated plasma samples for LBP and sCD14 concentrations at baseline, IRIS event or corresponding time point and at 3 and 6 months on treatment (Figure 2). We found significantly lower LBP values in TB-IRIS patients than in controls at baseline (p = 0.016, Figure 2A). In contrast, TB-IRIS patients showed significantly higher levels of LBP during IRIS event compared to controls (p = 0.010). In both groups, LBP levels increased after initiating ART, but this was much more pronounced for TB-IRIS patients. Over the next 6 months during ART, levels of LBP declined significantly in both groups and no longer showed significant differences between these two groups. No significant differences in sCD14 levels were apparent between TB-IRIS and controls at any time point (Figure 2B). Nevertheless, a significant increase of sCD14 was seen in TB-IRIS patients after initiation of ART compared to baseline (p = 0.037). 

### Cytokine levels prior to and during ART

The inflammation in TB-IRIS coincides with the production of a plethora of cytokines, which could result from TLR stimulation. To elucidate which cytokines take part in the development of TB-IRIS in our study, we analyzed plasma levels of 18 different cytokines. We compared values at baseline and during IRIS event or corresponding control time points between TB-IRIS patients and controls, as well as changes between both time points for each patient group (table 2). At baseline, we observed significantly lower cytokine levels in TB-IRIS patients than in controls for IL-6 (p = 0.041) and G-CSF (p = 0.036). During IRIS event, we observed significantly higher cytokine levels in TB-IRIS patients for IL-1RA, IL-4, IL-6, IL-7, IL-8 and G-CSF (p ≤ 0.032). These cytokines were also significantly increased at IRIS event compared to pre-ART in TB-IRIS patients. Additional increases over time were observed for IFNγ, CCL4 and CCL5 in TB-IRIS patients, while CCL4 and CXCL10 decreased over time in controls. Delta values (changes over time) of IL-6, IL-7, IL-8, G-CSF, CCL4, CXCL10 and LBP all showed significant differences between TB-IRIS patients and controls (p ≤ 0.028). IL-9 and IL-17 were below detection limit in >70% of our study subjects. No significant differences in detectability were observed between patient groups using dichotomous variables for both of these cytokines. IL-2, GM-CSF and TNFα were undetectable in >90% of our samples and were excluded from the analysis.

**Table 2:** Cytokines and other plasma markers in TB-IRIS patients and HIV+TB+ controls.

	**Baseline**			**IRIS event**			**Change over time (p^b^)**		**∆ change**
	**TB-IRIS (n=34^*a*^)**	**Controls (n=34^*a*^)**	**p^b^**	**TB-IRIS (n=31^*a*^)**	**Controls (n=31^*a*^)**	**p^b^**	**TB-IRIS (n=30^*a*^)**	**Controls (n=30^*a*^)**	**p^b^**
**Cytokines(pg/ml)**									
IL-1RA	56.7 (33.5-87.7)	54.1 (33.5-79.9)	0.831	89.3 (54.1-123.0)	46.6 (25.7-97.7)	**0.009**	**0.021**	0.558	0.054
IL-4	0.8 (0.2**^*c*^**-1.3)	1.1 (0.4-1.6)	0.217	1.2 (0.9-1.6)	1.0 (0.4-1.3)	**0.032**	**0.042**	0.882	0.094
IL-6	5.0 (2.9-8.3)	6.5 (3.7-13.0)	**0.041**	20.3 (10.4-43.7)	6.7 (3.0-11.6)	**<0.001**	**<0.001**	0.861	**<0.001**
IL-7	4.4 (3.5-5.1)	4.5 (3.5-5.3)	0.986	5.9 (3.8-7.4)	3.7 (3.1-5.8)	**0.017**	**0.008**	0.967	**0.021**
IL-8	7.7 (6.5-13.2)	9.0 (6.3-13.2)	0.614	10.4 (7-23.8)	7.3 (5.5-13.6)	**0.008**	**0.012**	0.171	**0.001**
IL-9	1.4 (1.4-1.4)**^*c*^**	1.4 (1.4-1.4)**^*c*^**	0.347	1.4 (1.4-1.4)**^*c*^**	1.4 (1.4-1.4)**^*c*^**	0.735	0.866	0.208	0.249
IL-10	2.4 (1.2**^*c*^**-6.0)	2.2 (1.2**^*c*^**-4.0)	0.302	4.5 (2.0-7.5)	1.2**^*c*^** (1.2**^*c*^**-6.5)	0.090	0.100	0.469	0.405
IL-12	2.2**^*c*^** (2.2**^*c*^**-4.4)	3.1 (2.2**^*c*^**-7.4)	0.355	4.4 (2.2**^*c*^**-7.9)	2.2**^*c*^** (2.2**^*c*^**-6.0)	0.236	0.126	0.614	0.501
IL-17	1.2 (1.2-1.2)**^*c*^**	1.2 (1.2-1.2)**^*c*^**	0.937	1.2**^*c*^** (1.2**^*c*^**-16.7)	1.2**^*c*^** (1.2**^*c*^**-24.5)	0.647	0.480	0.158	0.852
CCL11	79.7 (44.4-121.0)	102.9 (55.6-130.4)	0.122	72.5 (46.9-96.9)	75.1 (45.1-126.8)	0.614	0.770	0.050	0.465
G-CSF	29.7 (21.6-52.5)	40.7 (25.4-73.9)	**0.036**	45.6 (30.1-93.0)	44.3 (22.7-63.4)	**0.032**	**<0.001**	0.750	**0.003**
IFNγ	66.5 (29.9-103.0)	78.6 (46.9-132)	0.136	93.9 (58.8-129.3)	62.8 (39.1-138.4)	0.075	**0.028**	0.946	0.054
CCL4	47.9 (32.2-67.3)	66.6 (34.8-93)	0.092	71.8 (55.6-102.3)	65.4 (37.7-109.6)	0.456	**<0.001**	**0.001**	**0.028**
CCL5 (ng/ml)	4.4 (3.1-5.0)	4.3 (3.2-5.9)	0.437	5.5 (4.5-8.3)	5.8 (3.6-7.3)	0.170	**0.007**	0.271	0.178
CXCL10 (ng/ml)	8.4 (6.8-12.9)	10.3 (6.0-16.2)	0.726	11.4 (5.9-16.9)	5.5 (3.2-15.0)	0.057	0.125	**0.009**	**0.007**
**Other plasma markers**									
LBP **^*d*^** (µg/ml)	29.7 (15.42-37.1)	42.3 (25.1-53.5)	**0.016**	75.5 (45.0-≥100.0)	42.7 (27.13-70.4)	**0.010**	**<0.001**	**0.041**	**<0.001**
sCD14**^*d*^**(µg/ml)	3.4 (2.5-3.9)	3.3 (2.5-4.3)	0.342	3.6 (3.0-4.3)	3.5 (2.6-4.3)	0.879	**0.037**	0.940	0.192
I-FABP **^*d*^** (ng/ml)	1.0 (0.5-1.5)	0.9 (0.5-1.7)	0.741	0.6 (0.3-1.0)	1.1 (0.7-1.9)	**0.013**	**0.019**	0.355	0.113
EndoCab**^*e*^** (GMU/ml)	28.4 (20.7-59.0)	34.3 (18.2-61.7)	0.600	32.6 (17.6-59.8)	37.8 (15.6-65.2)	0.909	0.091	0.957	0.343
LPS **^*f*^** (EU/ml)	16.2 (11.5-19.5)	17.7 (15.3-20.4)	0.424	13.3 (11.4-14.2)	12.7 (11.9-16.2)	0.814	0.131	0.148	0.477

*Cytokine levels are shown as median values with inter-quartile range. Values were compared between matched patient pairs per time point and between time points per study group. Level of significance was set to p < 0.05.Differences between patients for IL-9 and IL-17 were analyzed with a McNemar test. IL-2 (LOD 2.0 pg/ml*)*, GM-CSF (LOD 20.4 pg/ml*)* and TNFα (LOD 8.8 pg/ml*)* were excluded from analysis.* **^*a*^** *Some patients were not included due to insufficient sample volume or pairwise exclusion.* **^*b*^** *Wilcoxon signed-rank test unless stated otherwise.* **^*c*^** *Values were below detection limit and represented as LOD/2.* ***^d^*** *Baseline n= 38, IRIS event n = 38.* ***^e^*** *Baseline n= 35, IRIS event n = 35.* ***^f^*** *Baseline n= 11, IRIS event n = 13. P-values in the final column represent the difference between delta (∆*)* values over time of TB-IRIS cases and controls*

### Central role of IL-6 during TB-IRIS

We next attempted to identify which of the cytokines that we established to be associated with TB-IRIS could lie at the basis of this cytokine storm. We thus explored which cytokines were interdependent or had independent effects. Plasma markers that showed significant (p < 0.05) univariate effects either pre-ART (IL-6 and GCSF) or during IRIS event (IL-1RA, IL-4, IL-6, IL‑7, IL-8 and G-CSF) were included in 2 separate multivariate conditional logistic regression models for paired patients. Only IL-6 was retained after stepwise elimination of covariates in the pre-ART model (p = 0.039, Odds Ratio: 0.87; 95% confidence interval: 0.77-0.99 per increase of 1 pg/ml) as well as in the IRIS event model (p = 0.034, Odds Ratio: 1.40; 95% confidence interval: 1.03-1.91 per increase of 1 pg/ml). We thus identified the innate cytokine IL-6 as the most central molecule in the cytokine disturbances occurring before and during inflammation in our TB-IRIS cohort.

## Discussion

In this study, we analyzed plasma levels of markers of increased intestinal permeability, PAMP-binding proteins as well as pro- and anti-inflammatory cytokines before and during ART in one of the largest TB-IRIS cohorts described to date. The role of myeloid cytokine patterns in TB-IRIS is becoming increasingly apparent [17]. Furthermore, PAMPs are well known inducers of myeloid cytokine responses through TLR stimulation [31] and PAMP-binding proteins can interfere with this induction [24]. Results from our study suggest that plasma IL-6, G-CSF and LBP levels prior to ART as well as IL-6, LBP and I-FABP levels during the first weeks of treatment are potential markers of TB-IRIS. In addition, we identified IL-6 as a key protein in the plethora of cytokines and chemokines we found to be associated with TB-IRIS. Our data thus support an important role for the innate immune system in TB-IRIS. 

Disease progression in AIDS patients is associated with increased immune activation, which has been linked to enterocyte damage and microbial translocation as a consequence of HIV infection and can persist during ART [32]. We hypothesized that a more pronounced presence of bacterial components such as LPS, which can enter the blood as a result of impaired intestinal barrier function in HIV patients, could enhance the inflammation in TB-IRIS patients. In this study, we report plasma LPS levels which are higher compared to studies that used endpoint detection methods of LPS instead of kinetic assays [33]. As comparing results across different platforms can be challenging and we report LPS levels in a limited number of TB-IRIS patients, these measurements should be confirmed in a larger cohort. Nevertheless, we observed no statistically significant differences in LPS levels between our study groups. While contrary to our hypothesis, this is in line with our findings on EndoCab and I-FABP. Our data therefore demonstrate no evidence that translocation of bacterial PAMPs through a leaky gut could have contributed to TB-IRIS in our study. In fact, we observed significantly lower levels of I-FABP in TB-IRIS patients during the 6 month follow-up period after ART initiation. I-FABP is specifically released into the bloodstream by damaged enterocytes and elevated plasma levels of I-FABP thus reflect damage to the intestinal epithelium. Though data on I-FABP in HIV patients during treatment are limited, higher I-FABP levels were found associated with impaired homing of T-cells to the gut (T-helper 17 cells in particular) [34], while lower I-FABP levels associated with better CD4+ T cell recovery [25]. Lower I-FABP levels during the first 6 months of treatment could thus reflect a stronger homing of T-cells to the gut, possibly as a reaction to elevated IL-6 levels which stimulate T helper 17 cell development. However, whether there is a causal contribution to TB-IRIS remains unclear. 

While a contribution of LPS to TB-IRIS thus seems unlikely, there is evidence of other PAMPs being implicated in IRIS pathogenesis. Indeed, higher cryptococcal antigen titers have been reported pre-ART in cryptococcal-IRIS [35], though it is unclear whether these are actual PAMPs. Moreover, the urinary concentration of LAM, a TB-associated PAMP, was found to be elevated in TB-IRIS patients from our cohort prior to the start of ART [7]. Mycobacterial PAMPs typically initiate the innate immune response to TB infection through stimulation of TLRs [36]. This response can be modulated by sCD14 and LBP, both of which can regulate binding of mycobacterial PAMPs to TLRs [37]. We thus hypothesized that these PAMP-binding proteins could regulate the inflammation in TB-IRIS by interfering with TLR stimulation by mycobacterial PAMPs. LBP is a well-known acute phase protein and as such we found it to be drastically upregulated during the ongoing inflammation at the time of TB-IRIS. Strikingly however, we found lower levels of LBP at baseline in TB-IRIS patients compared to control patients. It is tempting to speculate that these lower levels make TB-IRIS patients more sensitive to circulating LBP-binding PAMPs. Indeed, lower levels of LBP may be an indication of less antigen being cleared, leading to enhanced TLR stimulation and subsequent inflammation upon ART. Alternatively, lower levels of LBP could be a consequence of elevated mycobacterial PAMP concentrations, leading to the formation of antigen-LBP complexes which are bound to and/or internalized by CD14+ innate immune cells [24]. Thirdly, as LBP is produced by the liver in reaction to IL-6 [38], these findings could suggest a below average reaction to circulating PAMPs by the innate immune system in TB-IRIS patients before ART. 

Upon TLR stimulation, innate immune cells generally start releasing a number of cytokines with IL-6 as one of the first and central cytokines being produced [39], which eventually could lead to a phenomenon described as the cytokine storm during TB-IRIS [11]. We report elevated plasma levels of a number of cytokines commonly associated with activation of myeloid cells, such as IL-1RA, IL-6, IL-8, G-CSF, in TB-IRIS patients during the IRIS event. These results indicate a large contribution of the innate immune system during the inflammation of TB-IRIS, possibly as a result of TLR stimulation. Conversely, we observed lower IL-6 and G-CSF levels pre-ART, which points to perturbations in the innate immune system before ART has started. When including the fact that we observed lower LBP levels pre-ART, one could speculate that these lower cytokine levels reflect the inability of innate immune cells such as macrophages to mount a sufficient immune response to the presence of a large amount of PAMPs, as has been suggested previously [9,35,40]. Intriguingly, our multivariate models of cytokines that were significantly associated with TB-IRIS rendered the same innate molecule as the strongest predictor at both time points, narrowing down the plethora of cytokines that are associated with TB-IRIS to one single protein, IL-6. Interestingly, elevated IL-6 seems to be a recurring feature in most IRIS studies that included this cytokine [12,16,17], emphasizing its possible importance. Our findings during IRIS event are in agreement with two South African TB-IRIS studies [16,17], although these studies did not report pre-ART differences in TB-IRIS patients. However, a recent study of an Indian TB-IRIS cohort reported pre-ART elevations of IL-6 as a risk factor for TB-IRIS [18]. This difference can be explained by the stringent matching for baseline CD4 count which we applied to our patient selection. Furthermore, previous studies on TB-IRIS [40] and cryptococcal IRIS [35] reported lower pre-ART levels of the innate cytokines CCL2 and both TNFα and G‑CSF respectively, which is in line with our findings, although TNFα was undetectable in most of our samples. 

Taken together, our results support the theory that a disturbed function of the innate immune system during TB treatment sets the scene for TB-IRIS to occur after ART has been initiated. It is conceivable that a high PAMP burden is created upon TB treatment to which innate immune cells such as macrophages are not able to mount a sufficient immune response. Our findings of lower IL-6, G-CSF and LBP levels pre-ART are in line with this theory. Once ART has been initiated, the subsequent rise in CD4+ T-cells could thus provide the needed assistance to these innate immune cells to initiate a full blown acute phase response and give rise to TB-IRIS as was proposed previously [9]. While the significant increase in IFNγ between pre-ART and IRIS event in our study supports this, the fact that IFNγ levels during ART were not significantly different between our study groups still suggests that the innate immune system is responsible for the bulk of the inflammation during TB-IRIS, as is reflected by elevated IL-6 plasma concentrations among other innate cytokines. 

An important limitation to our study was the unpredictability of TB-IRIS, which poses a serious challenge to prospective studies to adequately match IRIS events to control time points. We matched IRIS events with samples from controls after 2 weeks or 1 month on ART with a margin of 1 week, yet still observed a statistically significant difference in timing. This difference results from IRIS events consistently occurring before matched control time points, with a median difference of 1 day. Furthermore, it cannot be excluded that in these resource limited settings, patients presenting with IRIS symptoms close to their next scheduled visit of 14 days might have waited a day or two before attending the clinic, introducing a potential bias in our reported timing of TB-IRIS. Nevertheless, our findings during TB-IRIS were overall consistent with other studies. Moreover, all IRIS events were diagnosed on the basis of clear inflammatory symptoms and were subsequently sampled, thus truly representing ongoing TB-IRIS related inflammation. 

In conclusion, we report lower plasma levels of LBP before ART initiation but higher levels of LBP during IRIS event. Similarly, the innate cytokine profile showed lower levels before ART and higher levels during TB-IRIS, with IL-6 holding a dominant role. Our results show no evidence of the possible contribution of a leaky gut to TB-IRIS and support the theory that dysfunctions in the innate immune system make a large contribution to TB-IRIS pathogenesis. Consequently, plasma levels of IL-6, LBP and I-FABP could be potential markers of TB-IRIS which should be validated in future clinical studies for the diagnosis and treatment of TB-IRIS.
